# Breast cancer in previously thyroidectomized patients: which thyroid disorders are a risk factor?

**DOI:** 10.2144/fsoa-2021-0029

**Published:** 2021-03-29

**Authors:** Giuseppa Graceffa, Gregorio Scerrino, Gabriella Militello, Iole Laise, Brenda Randisi, Giuseppina Melfa, Giuseppina Orlando, Sergio Mazzola, Calogero Cipolla, Gianfranco Cocorullo

**Affiliations:** 1 Department of Surgical Oncology & Oral Sciences, Unit of Oncological Surgery, University of Palermo, Via L Giuffré, 5, Palermo, 90127, Italy; 2 Department of Surgical Oncology & Oral Sciences, Unit of General & Emergency Surgery, University of Palermo, Via L Giuffré, 5, Palermo, 90127, Italy; 3 Unit of Clinical Epidemiology & Tumor Registry, Department of Laboratory Diagnostics, Policlinico P. Giaccone University of Palermo, Via L Giuffré, 5, Palermo, 90127, Italy

**Keywords:** breast cancer, thyroid autoantibodies, thyroid autoimmunity, thyroid carcinoma, thyroid disorders, thyroid dysfunctions

## Abstract

The aim of this study was to evaluate whether there are thyroid diseases in which breast cancer will appear later as well as the role of autoimmunity. This was a retrospective observational study. A total of 410 females (thyroid surgery and later breast cancer) and 524 females (thyroid surgery only) were compared with regard to pathological thyroid findings, thyroid hormones, thyroid autoimmunity and type of breast cancer. Thyroid autoimmunity, especially antithyroid peroxidase antibodies, significantly increased the risk of breast cancer (p < 0.01); however, this was not true for other thyroid diseases, including thyroid cancer. No variant of breast cancer was predominant, and only thyroid autoimmunity was associated with the risk of breast cancer. Further research is needed to explain the impacts of different antithyroid antibodies.

## Lay abstract

Several studies have long hypothesized a link between thyroid disease and breast cancer. The authors' study retrospectively examined a large cohort of patients who initially underwent thyroid surgery and subsequently had breast cancer compared with a control group consisting of patients with only breast cancer. This comparison showed that only autoimmune thyroid disease was a risk factor for subsequent breast cancer, whereas no significant association of thyroid cancer with breast cancer was found.

Breast cancer (BC) is the most frequent malignancy of the female sex worldwide. More than 2 million cases were diagnosed in 2018, mostly in developed countries, and particularly in Europe, where an incidence of 400,000 new cases and 98,000 related deaths were detected in the same year [1,2]. Relationships between endocrine system, metabolic disorders and BC, excluding the sphere of sexual hormones, have long been hypothesized, and many epidemiological and clinical studies have found significant correlations with diabetes, obesity and other endocrine disorders [3–6].

Thyroid dysfunction has also been found to be related to BC, with extremely conflicting results depending on the nature, methodology and objectives of the studies involved. A recent review analyzed studies concerning site-specific cancer risks associated with thyroid disorders (hyper- and hypothyroidism) [5]. Although some studies have shown a relationship between BC and hyperthyroidism, a meta-analysis did not lead to clear conclusions because of a lack of available data [7]. Nevertheless, some studies have suggested that the thyroid hormone alpha receptor acts as a signal inhibitor in BC [8] or, vice versa, as a MAPK pathway activator [9]. It has also been suggested that dietary iodine intake could induce cancer cell apoptosis [10]. A systematic review with meta-analysis of these data along with others did not demonstrate a clear association of thyroid dysfunction with BC; however, it did indicate, although with a low level of evidence, that strict hormonal control could help to reduce the risk of BC [11]. Several factors also seemed to influence the results of this study (data heterogeneity, confounders and limited size of samples of enrolled patients).

Some studies have addressed the association between BC and thyroiditis, with conflicting and often opposite results [12,13]. A recent case–control study carried out on a large number of patients with an initial diagnosis of BC, derived from a database, showed a significant epidemiological association between BC and thyroiditis, not related to other thyroid dysfunction [14]. A relationship between thyroid cancer (ThC) and BC has also been hypothesized, and some studies have demonstrated an epidemiological relationship between these cancers [15–17]. Genetic, environmental, hormonal and metabolic factors seem to explain this relationship [18].

In summary, studies that have been carried out so far on different aspects of the problem have not always been conclusive and, above all, are discordant. The relationship between ThC and BC is thus still largely uncertain. The aim of this study was therefore to examine, in two populations, both recruited in a surgical context, whether there are thyroid diseases in which it is more possible to observe, in a variable time span, the development of BC and whether autoimmunity plays a role in this regard.

## Methods

The present retrospective observational study was conducted on a sample of patients affected by BC who had undergone thyroid surgery for benign or malignant disease. In particular, the authors investigated whether there was a context of autoimmunity shown by antithyroid peroxidase antibody (ATPOAb) and/or antithyroglobulin antibody (ATGAb) assay and confirmed by histology.

The recruitment criterion was female patients undergoing surgery for BC from 1 January 2012 to 30 September 2019 with a history of thyroid surgery. Both surgical procedures were performed at one of the two surgical units (general and oncologic surgery, general and emergency surgery) at the authors' institution (University of Palermo, Palermo, Italy). The inclusion criterion was the availability of complete documentation for both thyroid and breast disease and related surgical procedures. Patients with a known history of further neoplastic pathology, arising before or between thyroid pathology and breast pathology or revealed during the minimum follow-up of 1 year, were excluded.

Initially, the number of female patients recruited for the study with thyroid surgery and later BC was 410. These patients came from the western provinces of Sicily (Palermo, Trapani and Agrigento) and had an average age of 45.7 years at the time of thyroid surgery and 59 years at the time of mastectomy. A group of 524 females, who had the same geographic origin and underwent only thyroid surgery during the same period, were selected with the same inclusion criterion as the control group. The average age of this group was 55.5 years.

The variables examined in both groups were age, type of thyroid surgery (hemithyroidectomy, total thyroidectomy) and definitive histological examination of the pathological thyroid findings (benign nodule or goiter = 1; goiter with thyroiditis = 2; Graves' disease = 3; carcinoma = 4). Thyroid autoimmunity was also reported as an autonomous variable, including both the antibody pattern and the pathological finding. Thyroid hormones (thyroid-stimulating hormone, free triiodothyronine, free thyroxine) and ATPOAb and ATGAb were also evaluated.

**Figure 1.**
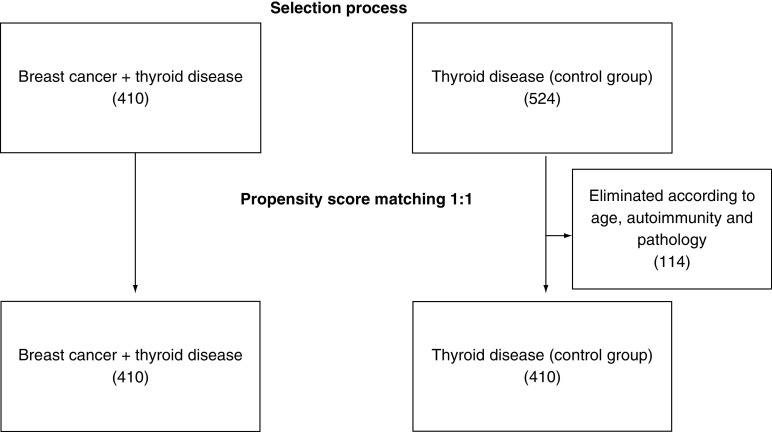
Propensity score matching process. Initially, the number of patients with thyroid surgery and later breast cancer was 410 compared with 524 patients with thyroid surgery alone. A 1:1 matching with the propensity score was performed to balance the group of cases with the control group according to the following covariates: age, autoimmunity (evaluated as a dichotomous variable) and histology.

In the group of patients with BC, the type of carcinoma found in the final pathological examination was also reported (ductal infiltrating carcinoma = 1; tubular carcinoma = 2; lobular carcinoma = 3). To eliminate the selection bias of observational studies, the group of cases and the control group were balanced by performing a 1:1 matching with the propensity score based on the following covariates: age, autoimmunity (intended as a dichotomous variable) and histology (Figure 1).

## Results

The univariate analysis (Table 1) showed that the two groups were not homogeneous, both in terms of number and autoimmunity, but especially in terms of age and histology distribution. The matching, as summarized in Figure 1, made the two groups more homogeneous based on the three aforementioned covariates. These two groups of equal number were balanced with regard to age, autoimmunity and pathology; however, there was still an imbalance in terms of pathology and autoimmunity (Table 2).

**Table 1.** Demographics and results.

Variable	Thyroid + BC	Control group	Total	OR (95% CI)	p-value
Age (years)	Average: 45.74	Average: 55.52			**2.2e-16**
Operation					
Thyroidectomy	378	500	878	1.76 (0.99–3.18)	0.05107
Thyroid lobe	32	24	56		
Total	410	524	934		
Pathology					
1	195	126	321		2.2e-16
2	155	135	290		
3	37	37	74		
4	23	226	249		
Total	410	524	934		
Autoimmunity					
No	192	279	471	1.29 (0.99–1.69)	0.05592
Yes	218	245	463		
Total	410	526	934		
Variable					
TSH (normal: 0.2–4.2 mlU/l)	Average: 2.22 mIU/l	Average: 2.45 mIU/l			**0.00515**
Variable					
FT3 (normal: 3.8–8.9 pmol/l)	6.14	6.08			0.6208
Variable					
FT4 (normal: 9–20 pmol/l)	14.61	14.69			0.6979
Variable					
ATPOAb	156.50	119.22			**0.001962**
Variable					
ATGAb	87.22	79.60			**0.03883**

Univariate analysis before matching.

Boldface values indicate statistical significance.

Pathology 1: Multi nodular goiter; 2: Hashimoto's thyroiditis; 3: Grave's disease; 4: Thyroid carcinoma.

ATGAb: Antithyroglobulin antibody; ATPOAb: Antithyroid peroxidase antibody; BC: Breast cancer; FT3: Free triiodothyronine; FT4: Free thyroxine; OR: Odds ratio; TSH: Thyroid-stimulating hormone.

**Table 2.** Demographics and results.

Variable	Thyroid + BC	Control group	Total	OR (95% CI)	p-value
Age (years)	Average: 45.74	Average: 45.50			0.7618
Operation					
Thyroidectomy	378	371	749	0.81 (0.48–1.35)	0.4564
Thyroid lobe	32	39	71		
Total	410	410	820		
Pathology					
1	195	221	416		0.01321
2	155	114	269		
3	37	53	90		
4	23	22	45		
Total	410	410	820		
Autoimmunity					
No	192	233	425	1.49 (1.12–1.99)	**0.01443**
Yes	218	177	395		
Total	410	410	820		
Variable					
TSH (normal: 0.2–4.2 mlU/l)	2.22	2.20			0.7796
Variable					
FT3 (normal: 3.8–8.9 pmol/l)	6.14	6.11			0.8581
Variable					
FT4 (normal: 9–20 pmol/l)	14.61	14.70			0.6514
Variable					
ATPOAb	156.50	94.35			**1.205e-07**
Variable					
ATGAb	87.22	68.60			**0.000294**

Univariate analysis after propensity score matching.

Boldface values indicate statistical significance.

Pathology: 1: Multinodular goiter; 2: Hashimoto's thyroiditis; 3: Grave's disease; 4: Thyroid carcinoma.

ATGAb: Antithyroglobulin antibody; ATPOAb: Antithyroid peroxidase antibody; BC: Breast cancer; FT3: Free triiodothyronine; FT4: Free thyroxine; OR: Odds ratio; TSH: Thyroid-stimulating hormone.

Based on the multivariate analysis (Table 3), adjusted using the autocorrelation between histology (autoimmunity), ATPOAb and ATGAb, histological autoimmunity resulted in a risk factor for BC at a later time. This was confirmed by the crossing of the different histological variants of BC with thyroid autoimmunity, which was treated as a dichotomous variable and, together with the antibody pattern, determined to be significantly associated (p < 0.01) with metachronous BC (Table 4). In addition, the authors can affirm that the increase of one unit of ATPOAb increased the probability of contracting BC after thyroid surgery by 11%, whereas an increase of one unit of ATGAb increased the risk of BC after thyroid surgery by 5%.

**Table 3.** Results

Variable	OR	95% CI (Inf)	95% CI (Sup)	p-value
Age	1.005	0.98	1.021	0.528274
Autoimmunity	15.62	3.73	57.54	**0.000103**
ATPOAb	1.113	1.09	1.143	**<0.0001**
ATGAb	1.046	1.03	1.058	**<0.0001**
Autoimmunity + ATAB	1.001	1.0006	1.0011	**<0.0001**

Multivariate analysis.

Boldface values indicate statistical significance.

ATGAb: Antithyroglobulin antibody; ATPOAb: Antithyroid peroxidase antibody; BC: Breast cancer; OR: Odds ratio.

**Table 4.** Cross-tabulation of the three different variants of breast cancer with the four groups of thyroid diseases evaluated.

Thyroid + BC	Control group	1	2	3	Total	p-value
1	221	159	12	24	416	0.07513
2	114	130	7	18	269	
3	53	33	1	3	90	
4	22	21	2	0	45	
Total	410	343	22	45	820	

Thyroid + BC = The four groups of patients with thyroid disease (multinodular goiter = 1; Hashimoto's thyroiditis = 2; Graves' disease = 3; thyroid carcinoma = 4).

BC: Breast cancer.

However, it should be noted that the average ATPOAb values in the case group and control group were 156.50 and 94.35, respectively. These values were far higher than the threshold of 35 IU/ml. By contrast, the ATGAb values of the two groups were significantly different, with values of 87.22 IU/ml (cases) and 68.60 IU/ml (controls), respectively, both of which were lower than the threshold of 116 IU/ml.

No correlation was found between variations in thyroid hormones and BC or, with the exception of autoimmunity, between the different thyroid pathologies and BC. In particular, no correlation was found between previous ThC and subsequent onset of BC, nor was any direct correlation found between Graves' disease and BC. Moreover, the overlap between histological variants of BC and different thyroid pathologies was not statistically significant (Table 5).

**Table 5.** Cross-tabulation of the three different variants of breast cancer with autoimmunity as a dichotomous variable.

Autoimmunity	Control group	1	2	3	Total	p-value
No	233	156	13	23	425	0.01694
Yes	177	187	9	22	395	
Total	410	343	22	45	820	

## Discussion

The hypothesis that there is some association between thyroid pathology and BC is quite widespread and very suggestive; however, the high frequency of both diseases creates a strong bias. For a long time, epidemiological studies have reported higher BC-related mortality in areas with a high incidence of goiter; conversely, the opposite has also been verified. The formulated hypothesis refers to effects on the mammary glands caused by thyroid dysfunction that is secondary, in turn, to ovarian dysfunction mediated by the pituitary gland [19]. Subsequent studies carried out on animal models have shown that the development of breast tissue is dose-dependent on exogenous L-thyroxine [20].

A more recent meta-analysis involving 13 selected case–control studies with the aim of investigating the association of hypothyroidism and hyperthyroidism with BC came to the conclusion that neither thyroid dysfunction nor the treatment implemented to correct it was correlated with an increase in the incidence of BC [11]. In particular, this result did not seem to be influenced by racial differences, and there was no correlation with pre- or postmenopausal age. However, the overall quality of the meta-analysis was low because of the observational nature of the studies included. The researchers also reported further limitations of the study, including the inability to assess risk factors such as alcohol and tobacco use, obesity and physical activity, all potentially related to BC; the case–control studies included, which were considered to have a risk of bias; and the total sample of patients enrolled, which was considered insufficient.

In a recent experimental study conducted on the cell lines MCF7, T47D, MDA-MB-468 and SKBR3, replacement hormone treatment was shown to be an independent variable associated with increased risk of BC in a subset of patients with estrogen-positive receptors and negative lymph nodes [21]. A moderate increase in BC risk was highlighted by a recent meta-analysis aimed at investigating the association between thyroid dysfunction and cancer incidence [7]. In this study, hyperthyroidism showed a pooled risk ratio of 1.20 (95% CI: 1.04–1.38) toward BC. However, the researchers stressed the lack of data regarding treatments and other confounders. These findings have an intriguing confirmation in the observation that reduced expression of type 3 deiodinase (a powerful peripheral inactivator of thyroid hormone) is associated with poor survival [22].

Having established that the data in favor of a relationship between thyroid dysfunction and BC are very weak, and that only hyperthyroidism could play a role, albeit a very doubtful one, the focus has shifted to the association of BC with metabolic syndrome. A meta-analysis based on 17 longitudinal studies with a minimum follow-up of 1 year evaluated the onset of BC during the follow-up of patients with metabolic syndrome compared with patients who were not affected [23]. The risk of BC was significantly increased in women of postmenopausal age but not in younger women, in which this condition reduces the risk of BC. The researchers declared potential bias, such as the simultaneous pharmacological treatment of some patients (e.g., with metformin), variability of criteria with regard to the definition of metabolic syndrome, absence of an interpretation of the causal relationship highlighted, reduced sample size of patients in some subgroups (e.g., certain races) and, finally, heterogeneity of the histological variants of BC included in different studies.

The prevalence of thyroid autoimmunity in patients with BC has been taken into account by some studies [24,25]. A prevalence of ATPOAb And ATGAb as well as chronic thyroiditis has been supported by several studies. An evident increase in BC risk in patients with autoimmune thyroiditis, with a pooled odds ratio of 2.92 (95% CI: 2.13–4.01), has been shown in one meta-analysis, with both ATPOAb and ATGAb responsible for this increase [26].

One retrospective study aimed to identify subgroups of patients with a higher risk of BC [27]. This study involved 867 patients, 140 with thyroid disease and BC and 726 with BC but without thyroid disease. The study demonstrated a strong association between BC diagnosed after menopause and thyroid disease (p < 0.003), and a significant association was also identified between premenopausal populations and chronic autoimmune thyroiditis (p < 0.05). Estrogen receptor expression was higher in patients with autoimmune thyroid disease compared with patients with other thyroid diseases.

A case–control analytical observational study conducted on 112 patients before or after surgery compared with 125 healthy patients showed only a greater representation of family history of thyroid disorders in the group of patients with BC. With regard to thyroid autoimmunity, it found a relationship with only marginal significance (p < 0.052) between estrogen receptor expression and thyroid autoimmunity [28]. More recently, a retrospective study showed a higher incidence of thyroid autoimmune disorders in a group of 793 patients [29]. These results were confirmed by a recent meta-analysis of raw data available in the literature (11 studies included) that showed higher ATPOAb and ATGAb values in patients with BC compared with subjects free of this tumor [30].

The relationship between thyroid autoimmunity and BC outcome has also been evaluated, but no evidence of a prognostic role for ATPOAb was found [31]. Other studies have shown that thyroid peroxidase can be weakly expressed by BC, so the presence of ATPOAb in these patients could be a consequence rather than a risk factor [32,33]. Finally, it has been demonstrated that the biochemical properties of breast- and thyroid-expressed thyroid peroxidase are similar [34].

With regard to the suggested common etiological context of BC and ThC, a recent study showed that women with BC have an increased risk with regard to the latter cancer [35]. In fact, the majority of epidemiological studies supporting this hypothesis, and which have shown up to a 67% increase in the risk of metachronous BC in patients with previous ThC, come from countries where much investment has been made in screening for the two cancers; this may be a significant bias [15,16,18,36,37]. Familiarity has also been put forward to explain a connection between both tumors. This can occur in some genetic syndromes related to ‘*PTEN*’ gene malfunctioning (i.e., Cowden, Bannayan-Riley-Ruvalcaba, Proteus-like). However, these are specific situations that do not explain, in all cases, the appearance of both cancers in the same patient [38–41].

From a more specific clinical point of view, a study conducted on a large population of patients undergoing surgical treatment for ThC did not show a significant increase in the risk of developing metachronous neoplasms in other sites, nor did ^131^I act as a risk factor in this context [42]. A recent review has also drawn controversial conclusions, depending largely on the type of setup, arguing that it is difficult to conduct multicenter, screening-based studies because of the risk of false positives for BC detectable in this context [43].

The authors of the current study confirmed the importance of thyroid autoimmunity as a risk factor for future BC. The strength of these results is evidenced by the double confirmation of autoimmunity, laboratory and histology. In addition, it was possible to highlight the preponderance of ATPOAb that increased considerably the risk of subsequent BC compared with ATGAb, whose effects were demonstrable but weaker. The association between increased antithyroid antibody pattern and risk of BC, as seen earlier, has already been noted in several published studies summarized in a review [30], but to date there is no comprehensive pathogenetic hypothesis. In the authors' study, the difference in impact between the two main antithyroid antibodies was shown for the first time. This finding may lead to a more comprehensive interpretation of why BC tends to occur more frequently in a context of autoimmunity and what mechanisms link this cancer to thyroid autoimmunity in particular.

The authors' study showed no association between thyroid diseases other than autoimmunity and BC. Grave's disease seems to play a role as autoimmune disorder, not because of the hyperthyroidism associated with it. Finally, no correlation was found between ThC and BC. Another strong point of the authors' study is the involvement of subjects in whom a histological diagnosis was always present. This allowed the authors to overcome any interpretative problem of autoimmunity, which was evaluated according to the most objective criterion possible. The origin of the patients from a well-defined geographical area is another strength of this study, as this aspect reduces the bias resulting from the possible influences of different environmental risk factors.

With regard to limitations, in addition to the retrospective nature of the study, it should be taken into consideration that, in spite of the balanced 1:1 propensity score, the distribution of pathology and autoimmunity remained unbalanced. Therefore, the persistence of a selection bias is possible. Another limitation of the study, largely determined by the recruitment of patients who had already undergone surgery, is the correction of thyroid hormone values at a stage prior to recruitment. This could make the values unreliable, as they are the result of prior treatment by the endocrinologists who referred these patients to us for surgery. Moreover, the authors must consider the inability to assess whether there is a correlation between levothyroxine-suppressive treatment and BC. This variable was not taken into account because during the period in which the study was carried out, there were different statements from the different endocrinologists and endocrine surgeons who treated the patients. Finally, risk factors such as alcohol and tobacco use, obesity and physical activity were not assessed. Despite these limitations, the authors believe that the large cohort of patients enrolled, evaluated over a sufficiently long period of time, with the same diagnostic standards and protocols is a value of the present study.

## Conclusion

The results of this study show that only autoimmunity is closely associated with the risk of developing BC over a variable period of time. The evidence of a significant difference in the impact of different antithyroid antibodies (ATPOAb vs ATGAb) suggests the need for further research, which should be developed from both a clinical and a biomolecular point of view. Thyroid dysfunction does not seem to have demonstrated, according to most of the literature, any role in the development of subsequent BC in patients with thyroid disease. Finally, there was no evidence of an association between thyroid cancer and BC in the present study. In this regard, the high frequency of both cancers, and in particular thyroid cancer, should be taken into account. In fact, the widespread investigation for thyroid cancer led to its overdiagnosis that has made this cancer much more common than in the past. Moreover, with regard to thyroid carcinoma, the very high long-term life expectancy associated with the disease should be taken into consideration, as the probability of randomly developing a further tumor will be higher, although this does not indicate a causal link. However, further studies will be useful in this regard to increase knowledge in this controversial field.

## Future perspective

In the near future, the concept of shared antigens between thyroid and breast tissues that trigger immune reactions in thyroid autoimmunity and BC will be explored in greater detail. In particular, we will understand more precisely whether thyroid autoimmunity (in terms of increased ATPOAb values), compared with BC, is a true risk factor, an occasional association or a consequence. From a clinical point of view, this may lead to the development of specific screening protocols targeted to subgroups of patients with thyroid autoimmunity.

**Summary points**

- Many epidemiological and clinical studies have found significant correlations of breast cancer (BC) with thyroid disorders.
- The authors compared a group of patients who had undergone thyroid surgery and then mastectomy for BC with a group undergoing thyroid surgery alone to investigate whether BC occurs at a higher frequency in patients with a history of certain thyroid diseases.
- The two groups were matched in terms of age, autoimmunity and pathology, and two groups of 410 patients were obtained.
- At multivariate analysis, antithyroid autoantibodies, in correlation with histological autoimmunity, were found to be risk factors for later BC.
- No correlation was found between variations in thyroid hormones and BC.
- Other than autoimmune thyroiditis, no correlation was found between different thyroid pathologies and BC. In particular, no correlation was found between previous thyroid carcinoma and subsequent onset of BC.
- Since a significant difference in the impact of different antithyroid antibodies (antithyroid peroxidase antibody greater than antithyroglobulin antibody) was found, further research is needed for a better interpretation of the pathogenetic correlations found.
